# Dissection of the Human T-Cell Receptor γ Gene Repertoire in the Brain and Peripheral Blood Identifies Age- and Alzheimer's Disease-Associated Clonotype Profiles

**DOI:** 10.3389/fimmu.2020.00012

**Published:** 2020-01-29

**Authors:** Maria Aliseychik, Anton Patrikeev, Fedor Gusev, Anastasia Grigorenko, Tatiana Andreeva, Arya Biragyn, Evgeny Rogaev

**Affiliations:** ^1^Department of Psychiatry, University of Massachusetts Medical School, Worcester, MA, United States; ^2^Department of Human Genetics and Genomics, Vavilov Institute of General Genetics, Russian Academy of Sciences, Moscow, Russia; ^3^Center for Genetics and Genetic Technologies, Faculty of Biology, Lomonosov Moscow State University, Moscow, Russia; ^4^Immunoregulation Section, National Institute on Aging, Baltimore, MD, United States

**Keywords:** Alzheimer's disease, T-cell receptor γ genes, immune repertoire, clonotype, immunogenic marker

## Abstract

The immune system contributes to neurodegenerative pathologies. However, the roles of γδ T cells in Alzheimer's disease (AD) are poorly understood. Here, we evaluated somatic variability of T-cell receptor γ genes (TRGs) in patients with AD. We performed deep sequencing of the CDR3 region of TRGs in patients with AD and control patients without dementia. TRG clones were clearly detectable in peripheral blood (PB) and non-neuronal cell populations in human brains. TRG repertoire diversity was reduced during aging. Compared with the PB, the brain showed reduced TRGV9 clonotypes but was enriched in TRGV2/4/8 clonotypes. AD-associated TRG profiles were found in both the PB and brain. Moreover, some groups of clonotypes were more specific for the brain or blood in patients with AD compared to those in controls. Our pilot deep analysis of T-cell receptor diversities in AD revealed putative brain and AD-associated immunogenic markers.

## Introduction

Low levels of chronic inflammation in aging, often termed “inflammaging,” are associated with immune dysregulation and represent a risk factor for decreased brain function and a hallmark of Alzheimer's disease (AD). Neuroinflammation occurs through activation of brain innate immune cells, such as microglia and macrophages, and further exacerbates the pathogenesis of AD (1). However, the roles of adaptive immunity and T cells in AD remain poorly understood. T cells can be induced by peripheral or brain antigen-presenting cells, such as microglia and macrophages, which take up and present antigens associated with AD. Animal modeling experiments have suggested that disease outcomes may depend on the type of brain-infiltrating T cells present in patients. Although regulatory T cells can be beneficial (2–4), human AD vaccine trials have been prematurely terminated due to induction of meningoencephalitis, presumably by infiltration of Aβ-specific Th1-skewed CD4 T cells (5, 6). In a rat model β-synuclein-specific T cells were found to invade gray matter, thereby inducing inflammation and neurodegeneration. β-Synuclein-specific T cells are also enriched in the blood of patients with multiple sclerosis (7).

The induction of antigen-specific T-cell responses is mediated by cell-surface T-cell receptors (TCRs). Upon somatic V(D)J recombination starting at γ- and δ- chains and then progressing to α- and β-chains, T cells are generated to express unique γδ- or αβ-TCR pairs on the cell membrane, respectively termed γδ and αβ T cells. Unlike αβ T cells, γδ T lymphocytes represent a less abundant population of peripheral blood (PB) cells (8) and have, on the one hand, diverse TCR repertoire (9), but, on the other hand, it is considered to be oligoclonal. For example, the top 20 clonotypes account for ~60% of all γδ T cells in the PB of adults (10). γδ T cells are also found in peripheral and barrier tissues, where they mediate antiviral and antimicrobial defense, support wound healing, and promote immune tolerance (11). γδ T cells can also be involved in pathological processes, such as oncogenesis (12), autoimmunity (13), and even stroke (14). These cells, particularly their interleukin (IL)-17 expressing subsets, have been shown to infiltrate lesions in the leptomeningeal envelopes after ischemic stroke. These cells are thought to have migrated from the intestine (14). Aging also differentially affects γδ T-cell subpopulations and their responses to infection. While young mice infected with West Nile Virus accumulate protective Vγ1 cells, which produce interferon (IFN)-γ to reduce viral infection and mortality from encephalitis, aged mice mostly increase tumor necrosis factor-α-expressing Vγ4 cells, which cause *blood-brain barrier (BBB)* permeability and thus brain infection and mortality (15). Neuroinflammation and consequent brain pathology appear to depend on a type of infiltrated γδ T cells. In mice with experimental autoimmune encephalitis (a model for human multiple sclerosis), Vγ4 cells produce various pro-inflammatory cytokines, including IL-17, whereas Vγ1 cells express CCR5 ligands and promote the differentiation of protective regulatory T cells (16). Human Vδ1 clonal expansion of γδ T cells in brain tissues is observed in Rasmussen's encephalitis (17). Despite the known roles of γδ T cells in neuroinflammation, their roles in AD are poorly understood.

The TCR γ gene (TRG) locus is rearranged in all T cells, and the CDR3 region of TRGs is a sensitive marker for T cells in cases where it is not possible to divide tissues into separate cell populations before DNA/RNA extraction. Furthermore, the small number of TRGV segments allows the use of a simple primer set that promotes all combinations of rearrangements with minimal amplification bias. Few T cells can penetrate brain tissues or may be found in the near-wall space of the vessels, perivascular space, meninges, and residual PB in the capillaries of the cerebral cortex.

In this study, by assuming that γδ T cells, as potentially regulatory or pathogenic cells, can infiltrate the brain, we sought to determine whether these cells were present in the brains of patients with AD. We compared the TCR repertoires of T cells from the PB and brains of patients with AD and patients without dementia using next-generation sequencing (NGS).

## Materials and Methods

### Blood and Brain Tissue Samples

The TRG repertoire was obtained for 163 PB and brain specimens, as described in the Supplementary Materials and Methods and Supplementary Table 1. DNA from PB and brain specimens was isolated using a Qiagen DNeasy Blood & Tissue Kit (Qiagen, Valencia, CA, USA). RNA from temporal and frontal cortex specimens was extracted using a Qiagen RNeasy Kit (Qiagen).

### cDNA Preparation

For reverse transcription and cDNA preparation, 1 μg RNA was used, and the reaction was carried out with a SuperScript First-Strand Synthesis System for RT-PCR (Invitrogen, Carlsbad, CA, USA). Random oligonucleotides were used as primers. The entire volume of the reaction mixture after completion of the protocol for reverse transcription was used to perform multiplex polymerase chain reaction (PCR).

### PCR and Methods for Obtaining the Repertoire of the CDR3 Region of Human TRGs

We developed a massive parallel sequencing TRG repertoire assay based on a set of oligonucleotides adopted from the BIOMED2 consortium, with modifications (18). Briefly, 200 ng genomic DNA (gDNA) or cDNA template was used for each PCR. The first reaction included primers for TCGV1/2/3/4/5/6/7/8 (TCGVf), TCGV10, and all TRGJ segments, whereas the second reaction included primers for TCGV9, TCGV11, and all TRGJ segments. Each reaction was repeated in duplicate. The oligonucleotides for TCR and the internal control are described in the Supplementary Materials and Methods. When gDNA was used as a template, oligonucleotides for amplification of a fragment of the internal control *ALB* gene were also added to the TCGVf/TCGV10 primer mix. For cDNA, oligonucleotides were used to amplify a part of the control *UBC* gene in separate tubes. We used PicoMaxx polymerase (Agilent, Santa Clara, CA, USA) for all amplification procedures. The TRG PCR conditions included 95°C for 2 min; 35 cycles of 95°C for 40 s, 60°C for 30 s, and 72°C for 40 s; and a final incubation at 72°C for 10 min. Preparation of libraries for massive parallel sequencing was carried out using a TruSeq DNA PCR-Free Library Prep Kit (Illumina, San Diego, CA, USA), followed by sequencing on the Illumina MiSeq platform. The average number of reads obtained per sample was 937,751 ± 379,308.

### Bioinformatics and Statistical Analyses

MiXCR program (19), version v2.1.5, was applied for TRG CDR3 repertoire extraction. These repertoires were filtered from non-functional CDR3 clonotypes (out of frame and stop codon-containing sequences), thereby significantly increasing the proportion of TRG rearrangements related to γδ T cells because ~70% of the rearranged TRGs in αβ T cells are not functional (20). According to rough estimates, ~30% of functional TRGs arise from γδ T cells in blood, and this percentage increases when analyzing the most abundant rearrangements, since the TRG repertoire in γδ T cells is more clonal (20).

CDR3 annotation, diversity analysis, and repertoire overlap analysis were performed using the VDJtools program (21), version v1.1.9. We used the VDJtools preprocessing correction module to eliminate clonotype pairs that differed by a specified number of mismatches (we used a threshold value of 2, which was rounded to the 0.9 percentile of the number of mismatches per 100 nt for each control gene sequence). When comparing TRG repertoires, we considered clonotypes as matching when they share the same amino acid sequences (CDR3aa only) in all analysis. We used VDJtools to determine the physicochemical parameters of the CDR3 regions (length, volume, hydropathy according to Kyte-Doolittle scale, strength), and we additionally calculated KF4 index for the AD-associated clonotypes analysis (Figure S6). In comparative brain vs. blood analyses of Vγ-segment usage, we used only the most frequent 20 clonotypes for each sample to adjust for unequal repertoire size. Statistical methods are described in Supplementary Materials and Methods.

## Results

### Method Validation

Because cell sorting cannot be applied to frozen PB and brain samples, we designed an approach based on a modified BIOMED2 consortium multiplex PCR system followed by library preparation and deep sequencing to characterize the TRG repertoires. Using this approach, we generated 165 libraries of the TRG CDR3 region from gDNA or RNA (cDNA) from the PB and brain frontal and temporal cortexes of 154 patients with neurodegenerative diseases and individuals without dementia (Supplementary Table 1). After filtering out reads with non-productive CDR3 (with stop codons or frameshift mutations), the average number of reads with productive rearrangements was 10^5^–10^6^ per sample. The average numbers of different clonotypes per sample found in the PB and brain cortex were 5 × 10^3^ (±2 × 10^3^) and 82 (±53), respectively.

We generated 439,049 clonotypes from PB and 6,872 clonotypes from brain samples. The total numbers for all samples of recurrent clonotypes were 220,986 in the blood and 2,340 in the brain (all clonotypes that were found in more than one individual were reported as recurrent). Moreover, 42.5% of all clones found in brain samples were also found in at least one blood sample.

First, we validated our methodology by analyzing independently processed sample duplicates (samples NL3 and A846). The results showed a high degree of similarity and the absence of significant PCR bias for the duplicated samples (Figures S1D,E). We also compared samples from different brain regions available from the same human subjects. Higher Jaccard coefficients (JCs) within repertoires of the frontal and temporal lobes from the same donor (average JC = 0.21 ± 0.099) were observed compared with other donors (average JC = 0.003 ± 0.0089). The results indicated a relative homogeneity and donor-specificity of the TRG repertoire in various areas of the cerebral cortex (Figure S1C).

Next, to directly confirm the presence of γδ T cells in human post-mortem brains, we utilized confocal microscopy analyses. Immunofluorescence staining clearly revealed the presence of T cells in cortical tissue sections from patients with AD and elderly individuals without dementia, where some cells were double positive for CD3 and γδ TCR (Figure S1F).

### TRG Repertoires Differed in the Brain and PB

**Figure 1:**
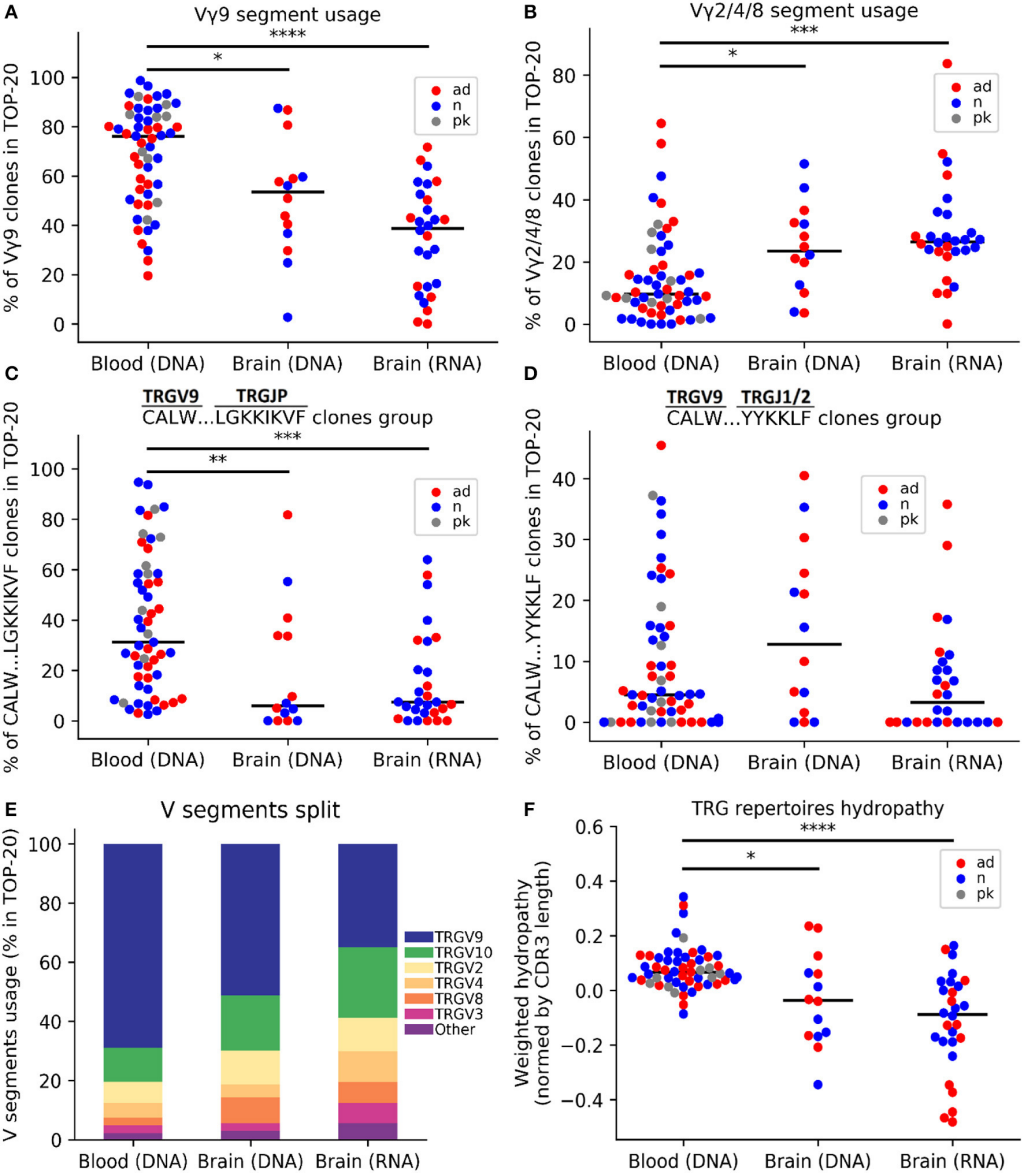
Comparative analysis of the TRG repertoire in the cerebral cortex and peripheral blood. The top 20 TRG repertoire the cerebral cortex differed in V-segment composition from that of the peripheral blood: the TRGV9 segment was predominant in the blood **(A)**, whereas the proportions of the TRGV2/4/8 segments significantly increased in the brain **(B)**. The TRGV9-TRGJP clone type “CALW…LGKKIKVF” (where dots indicate any set of amino acids) was significantly more frequent in the blood **(C)**, whereas other TRGV9 clones did not differ **(D)**. A schematic of the V-segment distributions of clones in different data groups is presented in **(E)**. The weighted average repertoire was more hydrophilic in the brain **(F)**. The colors denote various data groups: red, AD samples; blue, control samples; gray, patients with Parkinson's disease. Data were analyzed by Kruskal-Wallis ANOVA with Dunn's post-test comparisons, *P < 0.05, **P < 0.01, ***P < 0.001, and ****P < 0.0001.

For comparative analysis of TRG repertoires in the brain and PB, we analyzed samples from age-matched individuals. We compared frequencies of all V-segments found in top-20 clones repertoires: TRGV2, TRGV3, TRGV4, TRGV5, TRGV5P, TRGV7, TRGV8, TRGV9, TRGV10, TRGV11 (Supplementary Table 2) and TRGV2, TRGV4, and TRGV8 were combined because of their high similarity in nucleotide sequences. We found that cerebral cortex TRGV usage differed significantly from that in the PB (Figure 1E). For example, the frequency of the TRGV9 segment was the highest in the blood but significantly reduced in the brain (in terms of both DNA and RNA, *p* = 0.045 and *p* = 9.65e−08, respectively; Figure 1A). Moreover, combined usage of TRGV2/4/8 segments was lower in the PB (*p* = 0.028 and *p* = 0.0001 as compared with brain DNA and RNA, respectively; Figure 1B). We also noted similar differences when considered all available data instead of 20 most abundant clones (*p* = 0.0002 as compared TRGV9 frequency in PB vs. brain RNA; *p* = 0.008 and *p* = 0.009 as compared TRGV2/4/8 frequency in PB vs. brain DNA and RNA, respectively). Moreover, the different and common features of brain and blood samples were illustrated by specific clonotype groups. For example, we compared the frequency of the most known TRGV9-TRGJP public clonotypes group designated by us as CALW…LGKKIKVF (where dots indicate any number of any amino acids) which mostly included well-known germ-line CALWE(V)QELGKKIKVF public clonotype (20, 22), and also CALW…YYKKLF clones from the second most frequent TRGV9-J1/J2 group and also observed previously in the literature (23). Clonotypes of the CALW…LGKKIKVF group were more prevalent in the PB (*p* = 0.0096 and *p* = 0.0004 as compared with brain DNA and RNA, respectively; Figure 1C). But frequencies of clonotypes, identified by the sequence CALW…YYKKLF were similar in blood and brain samples (Kruskal test *p* = 0.16; Figure 1D). To further explore the differences between brain and blood TRG repertoires, we considered their chemical properties. We compared average CDR3 lengths (number of amino acids) and hydropathy indexes (according to the Kyte-Doolittle scale), volumes in Å^3^ (24), and strengths (interaction potential) (25), all of which were weighted according to the clonotype frequency and normalized to the CDR3 length, for brain and blood TRG profiles and found that repertoires in the brain were more hydrophilic (*p* = 0.016 for DNA and *p* = 7.757e−07 for RNA; Figure 1F). Importantly, the TRG repertoires from different brain regions (temporal and frontal cortex) of the same subjects showed similar patterns and individual specificity (Figure S1C). Together, these results indicated that the TRG profiles differed between the cerebral cortex and PB.

### Age-Related Changes in TRG Repertoire

**Figure 2:**
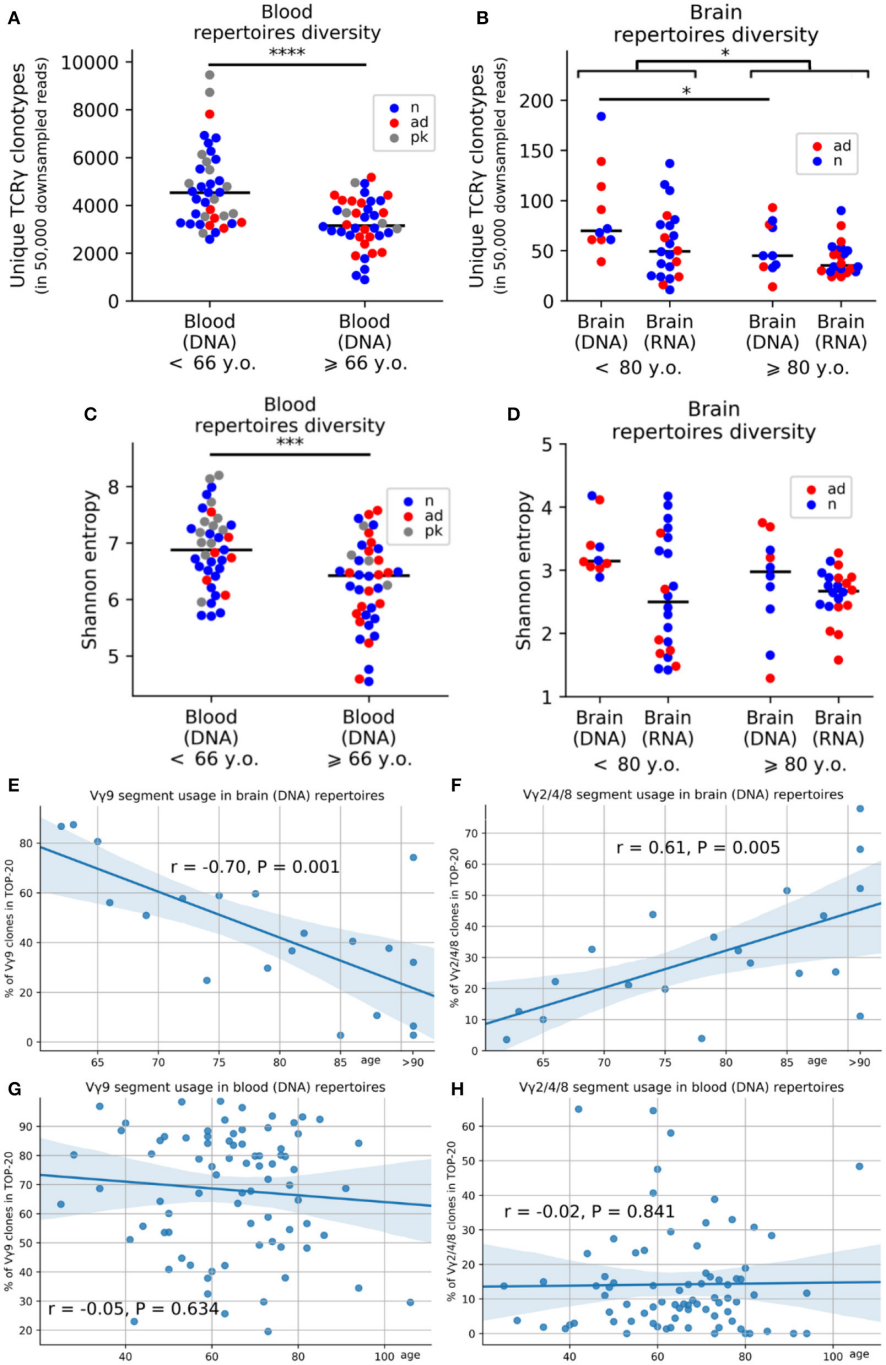
Age-related changes in the composition of the TRG repertoire in the blood and brain. Average numbers of unique clones after downsampling the data to 50,000 reads **(A,B)**. Changes in Shannon entropy with age in the peripheral blood **(C)** and brain **(D)** (red, AD samples; blue, control samples; gray, patients with Parkinson's disease). Regression analysis of V-segment frequencies in the top 20 clones for the TRGV9 segment **(E)** and TRGV2/4/8 segments **(F)** in the cerebral cortex and peripheral blood **(G,H)**. *P < 0.05, *** = < 0.001 and ****P < 0.0001.

To evaluate whether aging affected TRG profiles, we examine the PB (from individuals 25 to 106 years old) and brains (from individuals 21 years old to over 90 years old). The exact ages of individuals over 90 years of age for brain samples could not be obtained because of privacy reasons. We analyzed the effects of age by comparing samples from individuals above and below 80 years of age for brain samples and above and below 66 years of age for blood samples (these age thresholds were the median ages for brain and blood samples, respectively). Consistent with previous reports from other researchers (26, 27), we found that the repertoire diversity was decreased in the PB of aged individuals. After downsampling the data to 50,000 reads to account for unequal sequencing depth, we found, on average, 1.46 times fewer clonotypes in donors aged 66 years and older (*n* = 40, mean age = 76.65 years) compared with that in individuals younger than 66 years old (*n* = 39, mean age = 52.08; *p* = 4.424e−05; Figure 2A). This pattern remained statistically significant if we used only control individuals without dementia in the same analysis (*p* = 0.0007; Figure S2A). Regression analysis confirmed age-related reductions in the numbers of clonotypes in the PB (*p* = 7.004e−06). This age-related reduction in diversity was observed in group comparisons of brain samples but was not very pronounced (*p* = 0.026, Figures 2B,D; Figure S2B). Moreover, we estimated diversity using Shannon entropy and normalized Shannon-Wiener index as different metrics and obtained statistically significant age-related reduction of TCR diversity for TCR-rich PB samples [*p* = 0.0003 (Shannon entropy); Figure 2C, Figure S2C and *p* = 0.04 (normalized Shannon-Wiener); Figure S2D].

In addition to the age-dependent decreases in diversity, gDNA analysis of TRGV-segment usage revealed that blood-prevalent TRGV9 clonotypes were gradually decreased in the brain during aging (*p* = 0.001; Figure 2E), whereas brain-prevalent TRGV2/4/8 segments became more widely represented in brain of older individuals (*p* = 0.005; Figure 2F) but not in PB (Figure 2G–H).

### AD Was Accompanied by Accumulation of Clonotypes With Specific Amino Acid Properties

Finally, we investigated whether AD was associated with a unique TRG repertoire. Initial analysis of the chemical properties of total TRG repertoires did not reveal robust differences between AD and control samples (Figures S4A–H). We sought to identify a subset of clonotypes (subrepertoires) that differed between AD and control samples. Analyses of most represented TRGV9-TRGJP clonotypes did not reveal any significant differences (Figures S4I–P). Therefore, we mostly evaluated further non-TRGV9-TRGJP clonotypes. In this analysis, we excluded nine samples with a TRGV9-TRGJP clonotype frequency exceeding 40% as potential outliers (likely due to recent inflammation) (28), leaving 21 control and 20 AD samples for analysis of the PB and 29 control and 26 AD samples for analysis of the brain (all groups were age and sex matched; Supplementary Materials and Methods). We considered clonotypes shared (CDR3aa only) by at least two patients with AD, but not in any control samples, as “shared AD” and clonotypes shared by controls only as “shared norm”.

**Figure 3:**
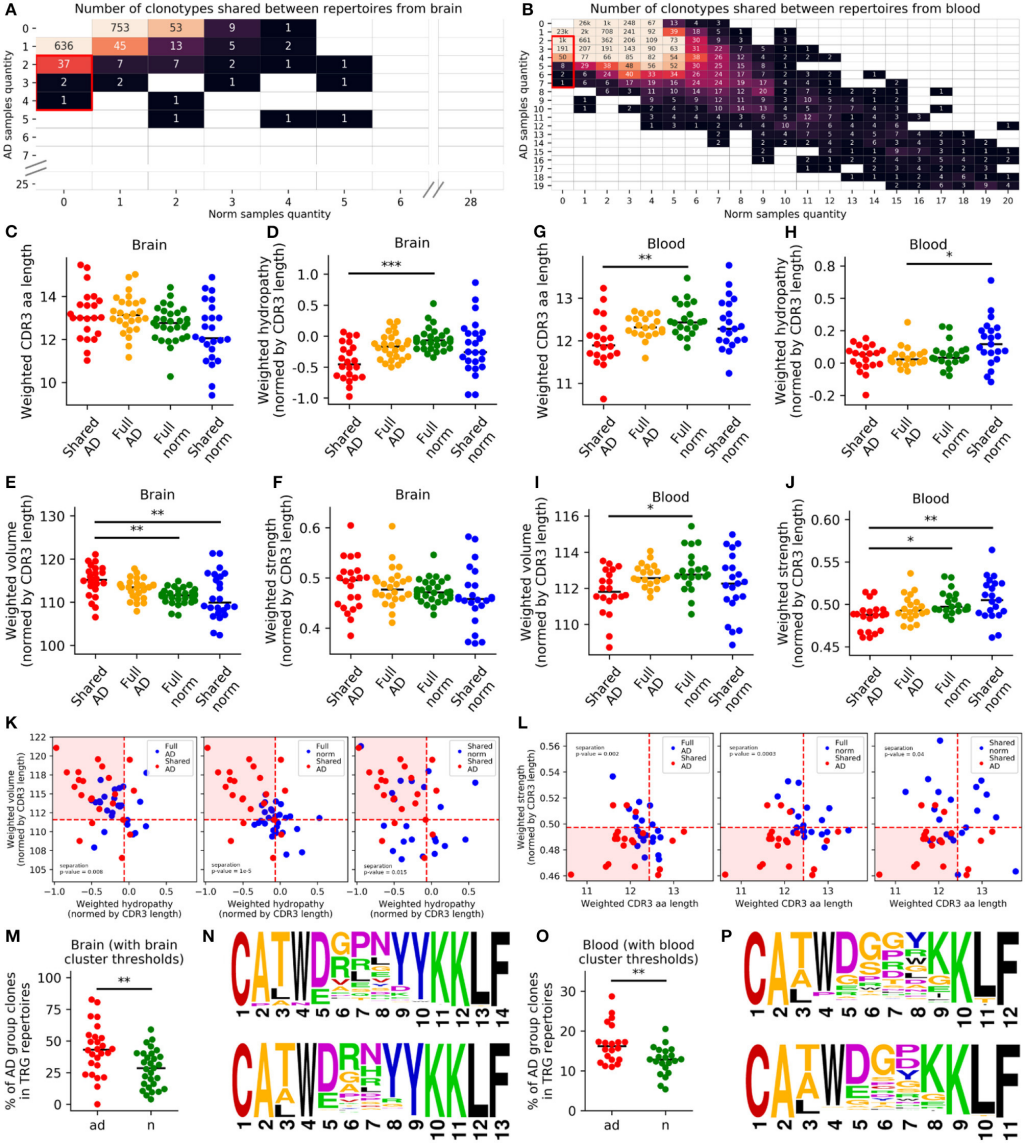
TRG repertoires in Alzheimer's patients included clones with specific properties in the blood or brain. We analyzed clones found in at least two patients, but not in healthy individuals **(A,B)**. Values in the matrices indicate numbers of clones found in the samples indicated along the axes. We compared the weighted CDR3 lengths **(C,G)**, hydropathy indexes **(D,H)**, volumes **(E,I)**, and strengths **(F,J)** between shared AD repertoires, full AD repertoires, total norm repertoires, and shared norm repertoires. For brain samples, values of volume and hydropathy were chosen **(K)**, and for blood, the CDR3 length and strength were chosen **(L)**, as characteristics defining AD-specific clone features. Clones with these characteristics were significantly more represented in patients with Alzheimer's disease **(M,O)**. Logoplots of CDR3 amino acid sequences for brain **(N)** and blood **(P)** were compiled (orange, small and weakly interacting amino acids; red, small; violet, small, weakly interacting, and hydrophilic; green, hydrophilic and weakly interacting; blue, hydrophilic). *P < 0.05, **P < 0.01, ***P < 0.001. Red dashed lines **(K,L)** denote median values for controls.

Using this approach, we found 40 shared AD clonotypes in brain samples and 1,255 shared AD clonotypes in PB samples (Figures 3A,B). Furthermore, we compared weighted average values of chemical properties (CDR3 length; hydrophilicity, volume, and strength normalized to CDR3 length) for shared AD vs. shared norm subrepertoires, full AD and full norm repertoires (excluding TRGV9-TRGJP clonotypes) for the brain and blood (Figures 3C–J). Average number of clones used for physical properties calculation for brain samples (Figure 3K) is 4.8 for shared and 76.4 for full repertoires; for blood samples (Figure 3L) it is 159.1 for shared and 4,487.5 for full repertoires. These group comparisons revealed that shared AD subrepertoires in the brain contained more hydrophilic and larger CDR3 regions than the other three subrepertoires (Figures 3D,E). Shared AD subrepertoires in the PB had shorter and weaker-interacting CDR3 regions (Figures 3G,J). In addition, cluster analysis of chemical properties (hydropathy indexes and volumes for the brain, CDR3 lengths and strengths for the PB) also demonstrated the significant segregation of shared AD subrepertoires from all other subrepertoires (Figures 3K,L, Figures S3A–D). We also made an analysis of the 5 central amino acids of the CDR3 regions only, which exhibits similar, but not so pronounced, trends (Figures S3G–L).

These initial observations suggested that the chemical properties of CDR3 regions may be altered in AD. To determine whether these results were applicable to the initial repertoires, we selected clonotypes with similar chemical properties using thresholds set to the median value of each property in the full normal repertoires (normalized hydropathy index <-0.066 and normalized volume >111.59 Å^3^ for brain samples; CDR3 length <13 amino acids and strength <0.497 for blood samples; Figures 3K,L). When comparing the frequencies of these clonotypes in the initial repertoires (which included TRGV9-TRGJP clonotypes as well) between patients with AD and age-matched controls, we found that the frequencies were significantly higher in patients with AD in both the brain (*p* = 0.003; Figure 3M) and the blood (*p* = 0.003; Figure 3O). Therefore, for both brain and blood tissues, we observed AD-associated clonotypes, as demonstrated in Supplementary Table 3 and Figures 3N,P.

Lastly, we used blood DNA from patients with Parkinson's disease (PK) as additional control to examine whether this AD-associated clonotype distribution was AD-specific or is observed also in other neurodegenerative diseases. After age-matching, we analyzed 18 patients with AD, 21 controls, and nine patients with PK. The described above clonotype signatures were detected in AD group only, but not in the PK group (Figure S5). Overall, these data imply that AD is characterized by accumulation of specific T-cell clonotypes.

## Discussion

To the best of our knowledge, in this study we present for the first time results of TCR repertoire profiling in patients with AD. We found that the TCR repertoire could be profiled using brain tissue samples. By focusing on the γ chain of the TCR, which occurs in γδ and αβ T cells (20), we found evidence of AD-associated enrichment of a unique TCR repertoire, presumably as a putative immunogenic signature. To this end, we combined next-generation sequencing with an in-house designed strategy that elucidated the diversity of TRG clonotypes in pools of genomic or transcriptome templates from whole peripheral blood and other human tissue compartments. Our bioinformatics approach for isolation AD-associated clonotypes was based on an analysis of TCRG rearrangements in patients with AD disease vs. individuals without dementia.

Notably, our method for TRG repertoire detection was robust in the human brain, however, the exact origins of these T cells have not been clarified. T cells could be derived from the intracerebral microvascular system or meninges of the brain (29) or from T cells infiltrating the brain parenchyma. Importantly, TRG repertoires from the human cerebral cortex were clearly distinct from those in the PB. Comparisons of top 20 most frequent clonotypes revealed robust differences in the frequencies of the TRGV9 segment and combined TRGV2, TRGV4, and TRGV8 segments in cortex vs. PB samples. Unlike TRGV9, the TRGV2/TRGV4/TRGV8 clones are more specific in the context of response to infection and are prevalent in non-blood peripheral body tissue compartments (30). Therefore, the brain repertoire is not merely a presentation of the vascular population of circulating blood cells. Antigen-specific T cells can cross the BBB and infiltrate the brain parenchyma when assisted by the MHCII molecule-expressing endothelium of cerebral vessels (31). Further studies, such as exploring the TCR repertoire in perfused mouse brains, may confirm our findings.

One important finding in this study was that the TRG repertoire in the cerebral cortex was influenced by age. We found age-related changes in the DNA repertoire in the brain, with brain-specific increases in the TRGV2/4/8 group. Moreover, the diversity of the TRG repertoire in the PB and brain was reduced in aged individuals, irrespective of their disease state, presumably reflecting the overall influence of inflammaging.

One obvious method for identifying disease-specific clones is to look for common clones in patient groups. However, the possibility of the formation of different CDR3 sequences to the same antigen and the incompletely elucidated mechanism of recognition of the antigen by γδ T cells, makes this approach insufficient. We analyzed clonotypes similar by several physicochemical properties, which may determine their specificity for similar antigens. AD is accompanied by accumulation of more hydrophilic residues and clones with larger volumes in the brain, and these features may indicate, for example, an immune response to lipids, which are known to present their hydrophilic portions to the CD1 molecule. The crystal structures of the complexes TCR with CD1 sulfatide (32) and CD1d α-galactosylceramide (α-GalCer) (33) were recently determined. Sulfatide belongs to sulfoglycolipids and is included in the composition of the myelin sheaths of neurons. Additionally, its content in the brain is disrupted at an early stage in patients with AD (34). α-GalCer is a synthetic glycolipid with a structure similar to sulfatide. Notably, all sequences of CDR3 clones specific to these antigens [α-GalCer, CATWDRGNPKTHYYKKLF (33); sulfatide, CATWDEKYYKKLF and CATWDRNNKKLF (32)] have AD-specific features in the brain, i.e., hydrophilicity and a larger CDR3 volume.

It must be elucidated further whether the AD-associated clonotype profiles are observed in various neurodegenerative and neuroinflammatory diseases. Self-antigens stimulating the γδ T cells immune response in AD might potentially share some signatures with a classic inflammatory disease of nervous system, such as multiple sclerosis. In this case, it may help to clarify the nature of the inflammation process in a course of AD. On other hand, our preliminary data indicate the uniqueness of the AD-repertoires, and, therefore, its potential use as biomarkers.

Overall, our data demonstrated the specific AD-associated clonotype features of TRGs derived from blood and brain cell populations. Further studies are needed to determine the relevance of cell subpopulations with such clonotypes to specific antigens. Our current findings highlighted potential new biomarkers for prediction and diagnosis of AD.

## Data Availability Statement

The datasets generated for this study can be found in the NCBI SRA database with SRA accession number PRJNA564281 and Supplementary Material (Data Sheet 2).

## Ethics Statement

The studies involving human participants were reviewed and approved by Institutional Review Board of the University of Massachusetts Medical School and Ethical Committee of Vavilov Institute of General Genetics RAS. The patients/participants provided their written informed consent to participate in this study.

## Author Contributions

MA carried out the experiments and wrote the manuscript. AP and FG conducted the bioinformatic and statistical data analysis. AG and TA supervised the DNA sequencing and corrected the manuscript. AB contributed to the discussion and editing of the manuscript. ER developed the study concept and wrote the manuscript.

### Conflict of Interest

The authors declare that the research was conducted in the absence of any commercial or financial relationships that could be construed as a potential conflict of interest.
