# Supplementary material for: Dissection of the Human T-Cell Receptor γ Gene Repertoire in the Brain and Peripheral Blood Identifies Age- and Alzheimer's Disease-Associated Clonotype Profiles
Source: Front Immunol. 2020 Jan 29;11:12. doi: 10.3389/fimmu.2020.00012 (PMC7025544; doi:10.3389/fimmu.2020.00012)
Supplement: Supplementary file 4 [file Data_Sheet_1.docx]

Supplementary Material

# Supplementary Materials and Methods

**1.1 Biological samples**

Biological samples, specifically, PB of patients with diagnosed Alzheimer's (23 samples/22 individuals) or Parkinson's disease (16 samples/individuals), as well as non-demented subjects (42 samples/41 individual), were collected after pre-signed informed consent (samples collected in Russian Federation (RF)). Samples of the frontal and temporal cortex were obtained from the US tissue banks: the New York brain bank (23 samples/individuals), the Oregon brain bank (27 samples/18 individuals), the Duke University tissue bank (25 samples/individuals), Maryland brain collection of National Institutes of Health NeuroBioBank (9 samples/individuals) (Supplementary Table 1). The samples with the short postmortem interval were used for immunofluorescent staining. The human brain specimens were originally frozen and stored without fixation and cryoprotectants, and deposition of lipofuscin was observed in the tissues of the elderly.

**1.2 The oligonucleotides for PCR assay**

The oligonucleotides TCGVf-5'-GGAAGGCCCCACAGCRTCTT-3', TCGV10-5'-AGCATGGGTAAGACAAGCAA-3', TCGV9-5'-CGGCACTGTCAGAAAGGAATC-3', TCGV11-5'-CTTCCACTTCCACTTTGAAA-3', TCRGJ1.2/2.1-5'-TTACCAGGCGAAGTTACTATGAGC-3', TCRG-J1.3/2.3-5'-GTGTTGTTCCACTGCCAAAGAG-3', TCRGJP-5'-CTGTAATGATAAGCTTTTTCCGG-3' were used to amplify the CDR3 of human TRG regions. ALBfor-5'-TGAACAGGCGACCATGCTT-3, ALBrev-5'-CTCTCCTTCTCAGAAAGTGTGCATAT-3', UBCfor-5'-CGGCCTTAGAACCCCAGTAT-3' and UBCrev-5'-TCACGAAGATCTGCATTGTCA-3'oligonucleotides were used to amplify the internal control genes.

**1.3 Immunohistochemistry**

Cerebral cortical sections (10–12 µm thick) were air dried and fixed in cold paraformaldehyde 4% for 15 min. After washing, sections were incubated in TrueBlack (Lipofuscin Autofluorescence Quencher; Biotium, Fremont, CA, USA) for 1 min. For blocking, normal serum block (NSB) reagent (Biolegend, San Diego, CA, USA) was used. Sections were incubated with primary antibodies for 3 h at 4°C, at room temperature for 30 min, and then with secondary antibodies in NSB for 1 h at room temperature. Sections were dried completely and coated with ProLong Diamond Antifade Mountant with DAPI (Thermo Fisher Scientific, Waltham, MA, USA). The day after staining, samples were analyzed by confocal microscopy. For immunofluorescent staining polyclonal rabbit Anti-CD3, affinity isolated, "DAKO" (1: 100); Anti-TCR gamma / delta monoclonal antibodies (5A6.E9) mouse IgG1 "ThermoFisher" (1: 100); Goat anti-Mouse IgG (H + L) highly cross-adsorbed secondary antibodies conjugated with Alexa Fluor 488 "ThermoFisher" (1: 1000); Goat anti-Rabbit IgG (H + L) cross-adsorbed secondary antibodies conjugated with Alexa Fluor 568 "ThermoFisher" (1: 1000); Purified mouse IgG1, κ Isotype control "Biolegend"; Purified rabbit polyclonal isotype control antibodies "Biolegend" were used.

**1.4** **Bioinformatics and statistical analyses**

We used VDJtools correction and decontamination modules for initial repertoires pre-processing. For further analysis we have also filtered clonotypes with CDR3aa sequence length above 27 amino acids as rare PCR artifacts.

Bioinformatics and statistical analyses were conducted using in-house bash and Python 3 scripts. Normality assessment for each dataset was performed using Shapiro-Wilk tests. Repertoire properties obtained from different samples were compared using two-tailed Student’s t-tests (for cases of unequal variance) for normally distributed data and Mann-Whitney test for nonparametric data. For comparison of repertoire properties obtained from one donor (comparison of repertoires from temporal and frontal lobes of the brain), Student’s t-tests for dependent samples were used in cases of normally distributed data, and Wilcoxon tests were used for nonparametric data. Multiple group analyses were performed using one-way analysis of variance (ANOVA) with Dunn’s post-hoc tests for normally distributed data or with Kruskal-Wallis with Dunn’s post-hoc tests for nonparametric data. Holm correction was applied for multiple testing.

For correlation analysis, Pearson’s correlation coefficient was used for normally distributed data, and Spearman correlation was used for nonparametric data. The Morisita-Horn similarity index was used to illustrate the similarity of repertoire replicas.

For comparative analysis of TRG repertoires in PB and brain, we analyzed samples from the age-matched subjects for peripheral blood DNA (n=55, female/male/unknown = 29/25/1, age 55-86, mean age=69.73), cerebral cortex DNA (n=14, female/male = 5/9, age 62-86, mean age=74.07) and cerebral cortex RNA (n=28, female/male = 11/17, age 57-86, mean age=73.57). All group comparisons were performed using Kruskal-Wallis with Dunn’s post hoc tests with Holm correction for multiple testing.

To analyze age effects on TRG repertoires we compared samples groups above and below median age for brain and blood sets: blood samples below 66 years old (n = 39, female/male = 26/13, age 25-65, mean age = 52.08) and 66 years old and older individuals (n = 40, female/male/unknown = 19/20/1, age 66-106, mean age = 76.65); brain from DNA samples below 80 years old (n = 10, female/male = 4/6, age 62-79, mean age = 70.3) and 80 years old and older individuals (n = 10, female/male = 5/5, age 81-93, mean age = 88.53); brain from RNA samples below 80 years old (n = 22, female/male = 9/13, age 21-76, mean age = 61.77) and 80 years old and older individuals (n = 22, female/male = 10/12, age 80-90, mean age = 86.17). Data on graphs A, B and D analyzed by two-tailed Student’s test; data on C graph analyzed by Mann-Whitney test. Correlation was assessed with Pearson’s correlation coefficient for E, F data and Spearman’s coefficient for G, H data.

For Alzheimer’s disease specific amino acid properties of TRG repertoires we compared control and AD samples from PB (AD: n = 20, female/male = 10/10, age 57-82, mean age = 72.25; controls: n = 21, female/male/unknown = 10/10/1, age 55-81, mean age = 68) and from brain (AD: n = 26, female/male = 13/13, age 62-93, mean age = 79.32; controls: n = 29, female/male = 12/17, age 61-83, mean age = 69.75). Data on graphs C, E, F, G and I analyzed using one-way ANOVA with Dunn’s post-tests; data on graphs D, H and J analyzed by Kruskal-Wallis with Dunn’s post hoc tests; K and M data was analyzed using two-tailed Student’s test.

To analyze age effects on TRG repertoires of non-demented individuals we compared samples groups above and below median age for brain and blood sets: blood samples below 66 years old (n =21, female/male = 14/7, age 25-65, mean age = 50.29) and 66 years old and older individuals (n = 20, female/male/unknown = 8/11/1, age 66-106, mean age = 77.15); brain from DNA samples below 80 years old (n = 4, female/male = 2/2, age 63-78, mean age = 70.25) and 80 years old and older individuals (n = 6, female/male = 2/4, age 81-93, mean age = 89,195); brain from RNA samples below 80 years old (n = 16, female/male = 5/11, age 21-76, mean age = 59.38) and 80 years old and older individuals (n = 10, female/male = 4/6, age 80-90, mean age = 86.66). Data on graphs A and C analyzed by two-tailed Student’s test; data on B and D graph analyzed by Mann-Whitney test. Correlation was assessed with Pearson’s correlation coefficient for E, F data and Spearman’s coefficient for G, H data.

Cluster analysis of chemical properties of shared AD subrepertoires from all other subrepertoires (Figure 3K, L and Supplementary Figure 3A–D). Each graph consists of two parts: scatter plot of two pre-selected amino acid properties (each dot is a sample with corresponding values) and histogram of average distances between clusters and statistical significance of observed distance (depicted by red dashed line and text) obtained using permutation testing approach. Using this approach, we iteratively (n=10000 iterations) randomly shuffled sample labels and calculated average distance between samples with different labels and therefore generated a distribution of such distances. Then we calculated a probability of getting between cluster distance bigger than observed (p-value showed on each second graph).

We compared frequencies of Alzheimer’s disease specific clonotypes groups between age-matched AD (n = 18, female/male = 9/9, age 57-80, mean age = 71.17), control individuals (n = 21, female/male/unknown = 10/10/1, age 55-81, mean age = 68) and individuals with Parkinson’s disease (n = 9, female/male = 7/2, age 57-78, mean age = 65.44). Data on graph analyzed by Kruskal-Wallis with Dunn’s post hoc tests.

# Supplementary Figures


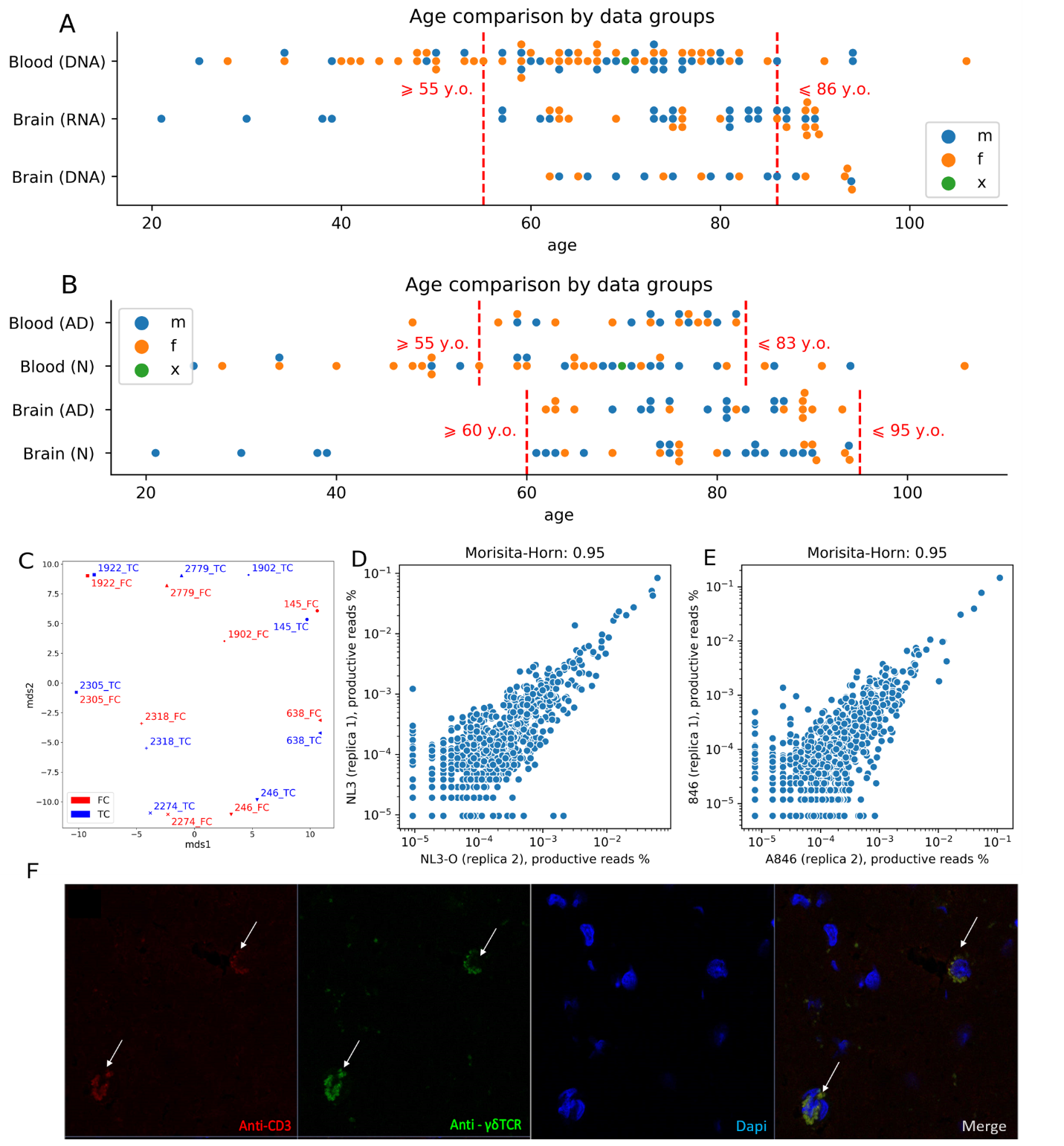


Fig. S1. **Age-matched cohorts and method validation.** We used an age filter from 60 to 86 years old comparing TRG data obtained from blood DNA, brain DNA, and brain RNA samples (A). We used an age filter from 55 to 83 or from 60 to 95 years old to compare the AD group with the control group according to data from the blood and brain, respectively (B). Blue color indicates male (m), orange color indicates female (f), green color is unknown gender (x). Multidimensional scaling analysis showed that TRG repertoires from the frontal and temporal cortexes of the same individual were clustered together using Jaccard indexes (C). Independent replicas of two peripheral blood TRG repertoires demonstrated a high degree of reproducibility (D, E). Confocal microscopy confirmed the presence of gamma-delta T cells (arrows) in sections of the human brain (F).


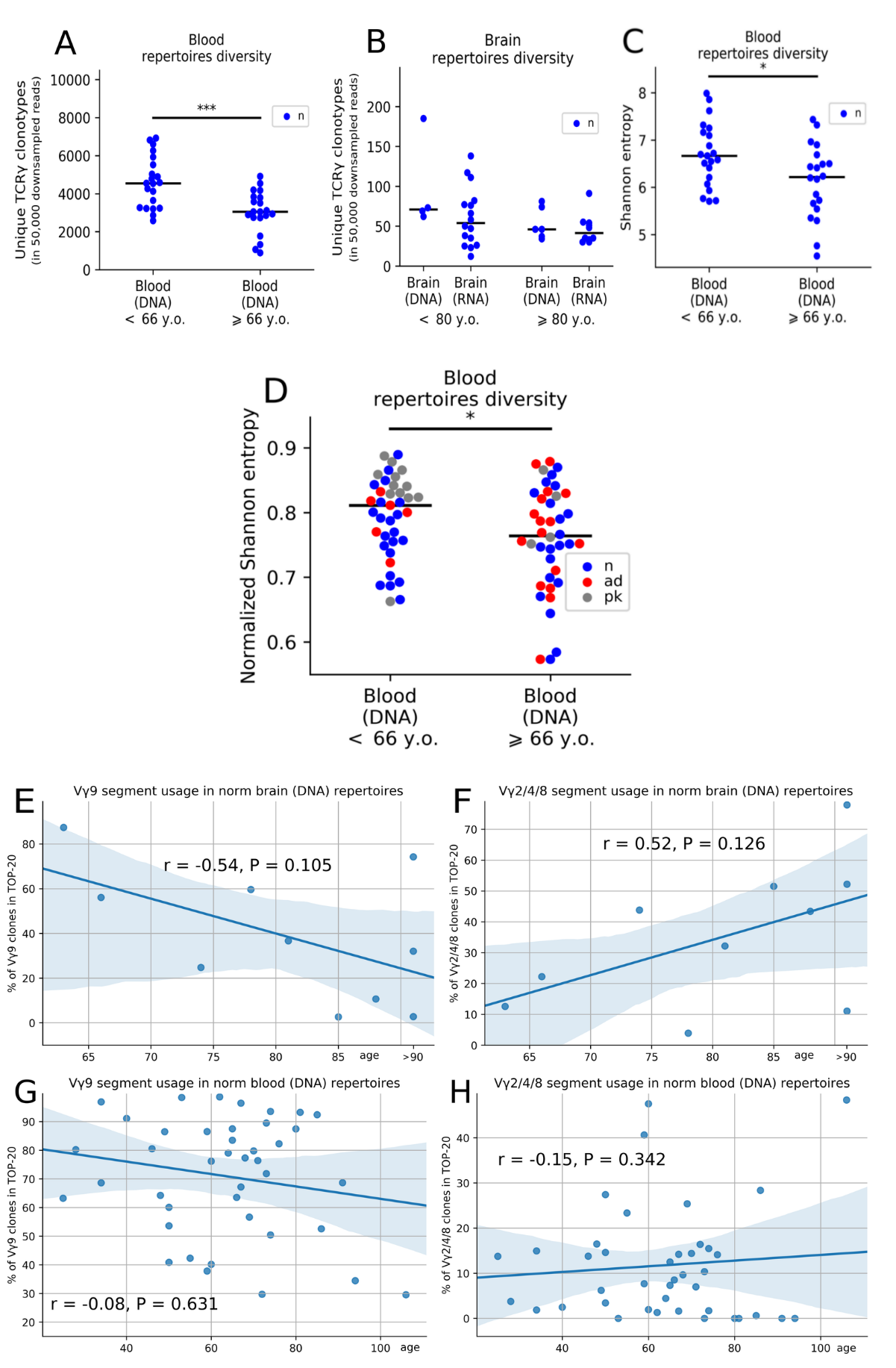


Fig. S2. **Age-related changes in the composition of the TRG repertoire.** The average number of unique clones after downsampling the data to 50,000 reads was reduced in the normal group of older people among all data types (A, B); however, this reduction was statistically significant only in the PB (A). We also tested separately TCR repertoire from PB using both Shannon (C) and Shannon-Wiener (normalized Shannon) (D) entropy. Both methods provide a similar result showing statistically significant age-related reduction of TCR diversity for TCR repertoire-rich PB samples. Regression analysis of V-segment frequencies in the top 20 clones of control repertoires, showing a decrease in the TRGV9 segment (E) and increase in the TRGV2/4/8 segments (F) in the cerebral cortex, but not in the peripheral blood (G, H). *P < 0.05, **P < 0.01, ***P < 0.001.


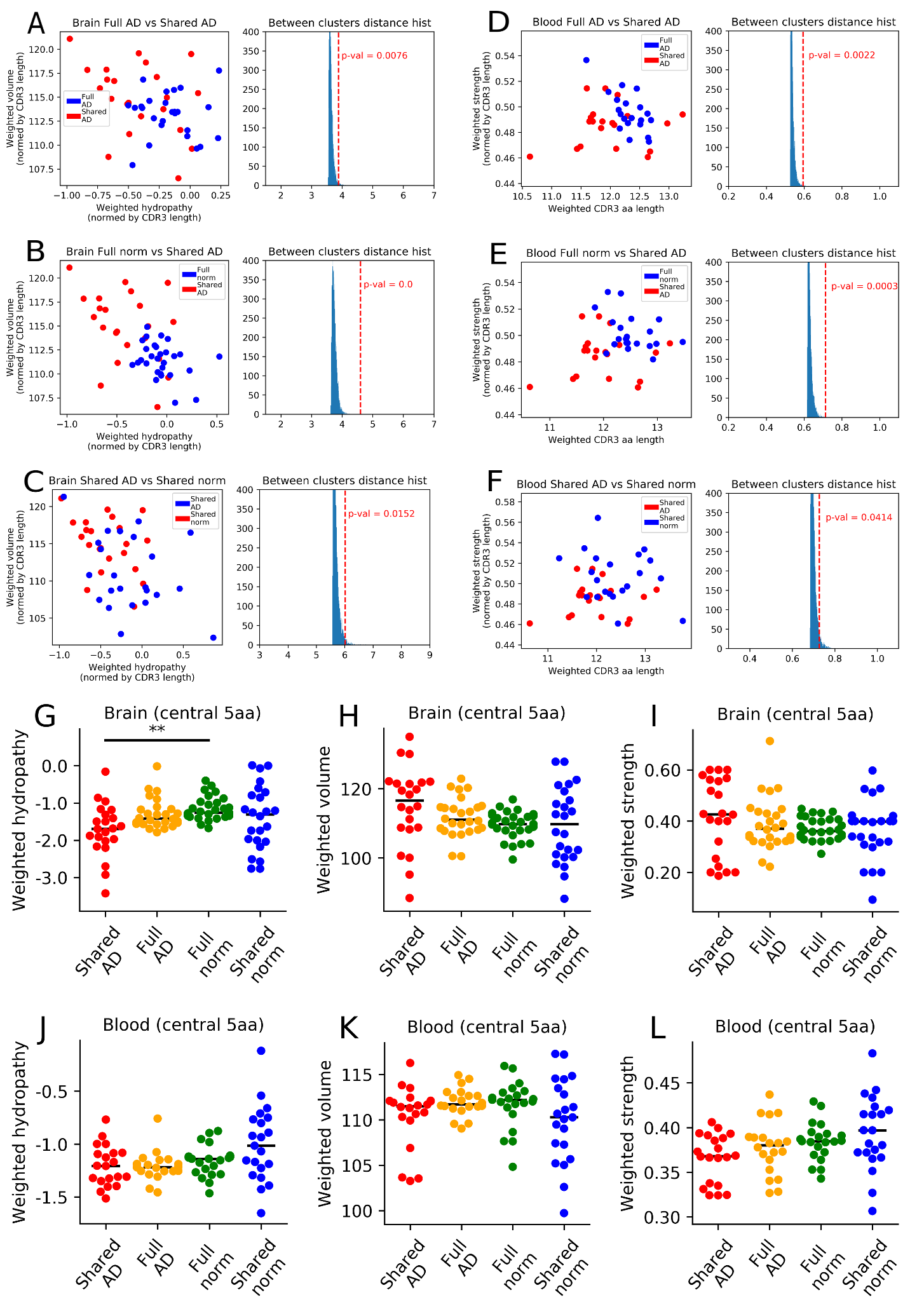


Fig. S3. **Visualization of random permutation tests and CDR3 central 5aa analysis.** We demonstrated the statistical significance of shared AD subrepertoire cluster separation using a random permutation testing approach (A-F) (see Supplementary Materials for more details). Each graph consisted of two parts: a scatter plot of two preselected amino acid properties (each dot is a sample with corresponding values) and a histogram of average distances between clusters (red dashed lines correspond to the observed distances between clusters, and numbers near these lines indicate *P* values for n = 10000 permutations as a significance measure of cluster separation). We additionally compared the weighted CDR3 hydropathy indexes (G, J), volumes (H, K), and strengths (I, L) for 5 central amino acids between shared AD repertoires, full AD repertoires, total norm repertoires, and shared norm repertoires from brain (G-I) and blood (J-L).


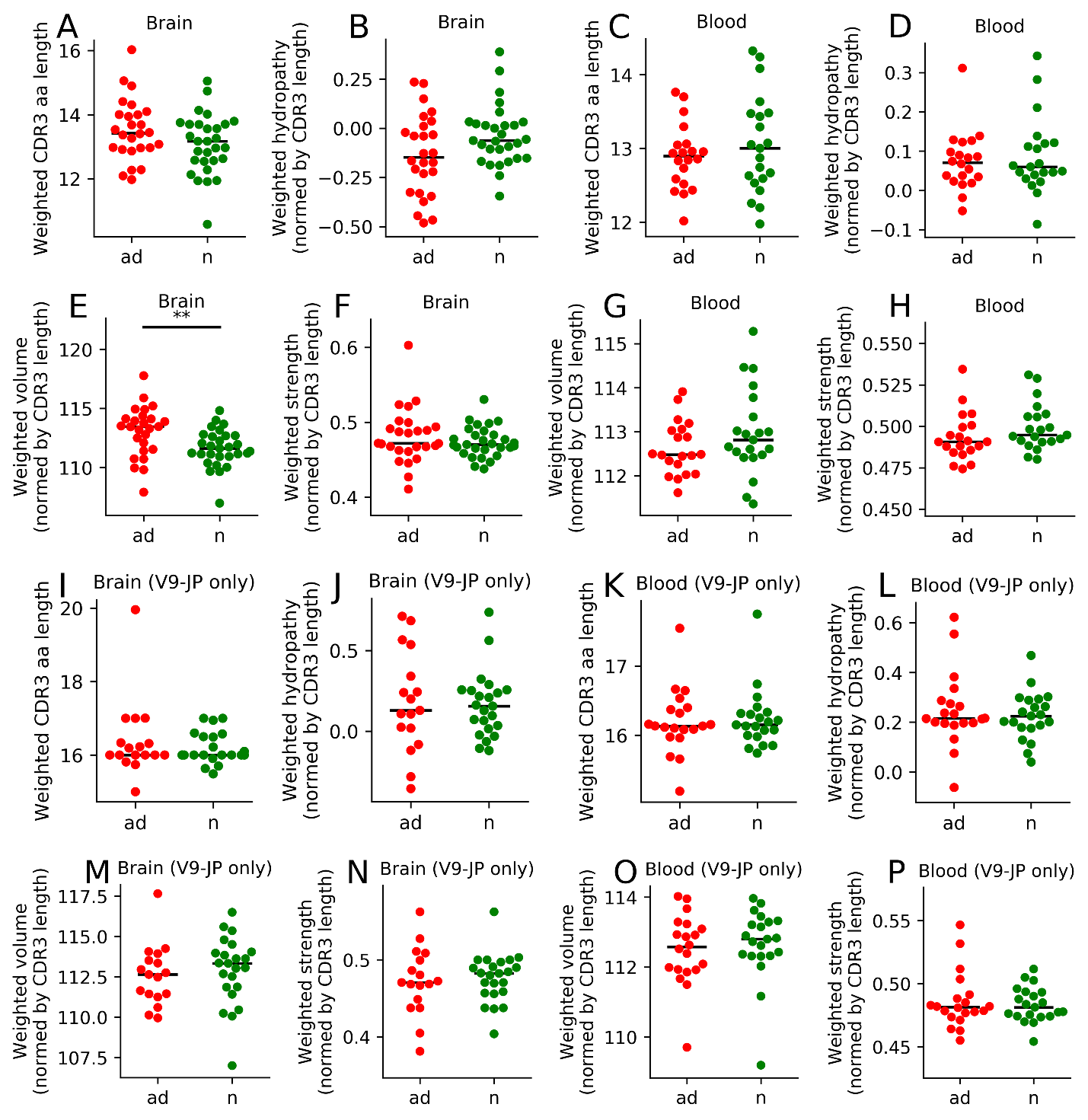


Fig. S4. **Comparison of initial repertoires (A-H) and V9-JP subrepertoires (I-P) amino acid chemical properties between AD (ad) and non-demented control (n) groups**. We compared the weighted CDR3 lengths (A, C, I, K), hydropathy indexes (B, D, J, L), volumes (E, G, M, O), and strengths (F, H, N, P) between AD and norm repertoires (A-H) or between AD and norm V9-JP subrepertoires (I-P); ***P* < 0.01.


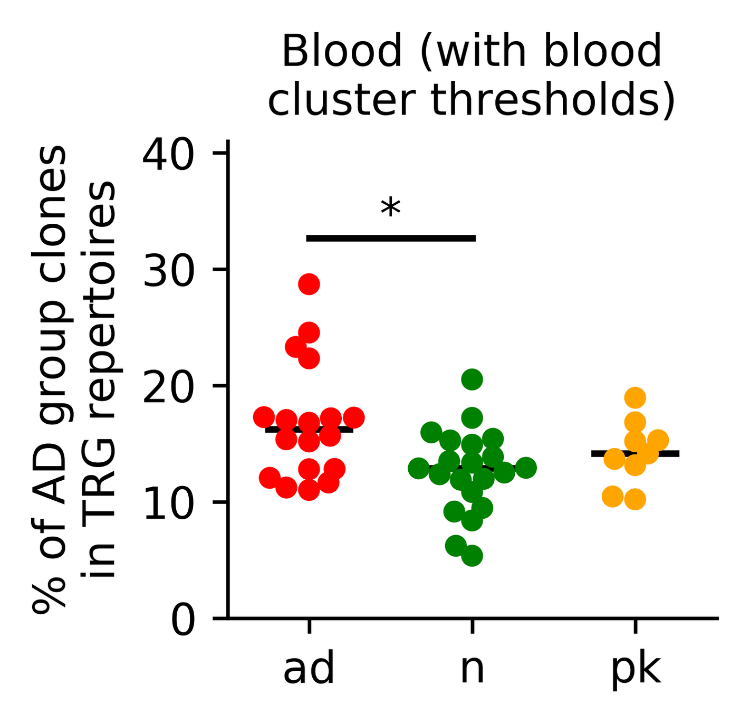


Fig. S5. **Frequency comparisons for subrepertoires with AD-specific amino acid chemical properties for age-matched groups of individuals with AD, non-demented control subjects (n) and Parkinson’s disease (pk).**The colors denote various data groups: red, AD samples; blue, control samples; yellow, patients with Parkinson’s disease. *P < 0.05.


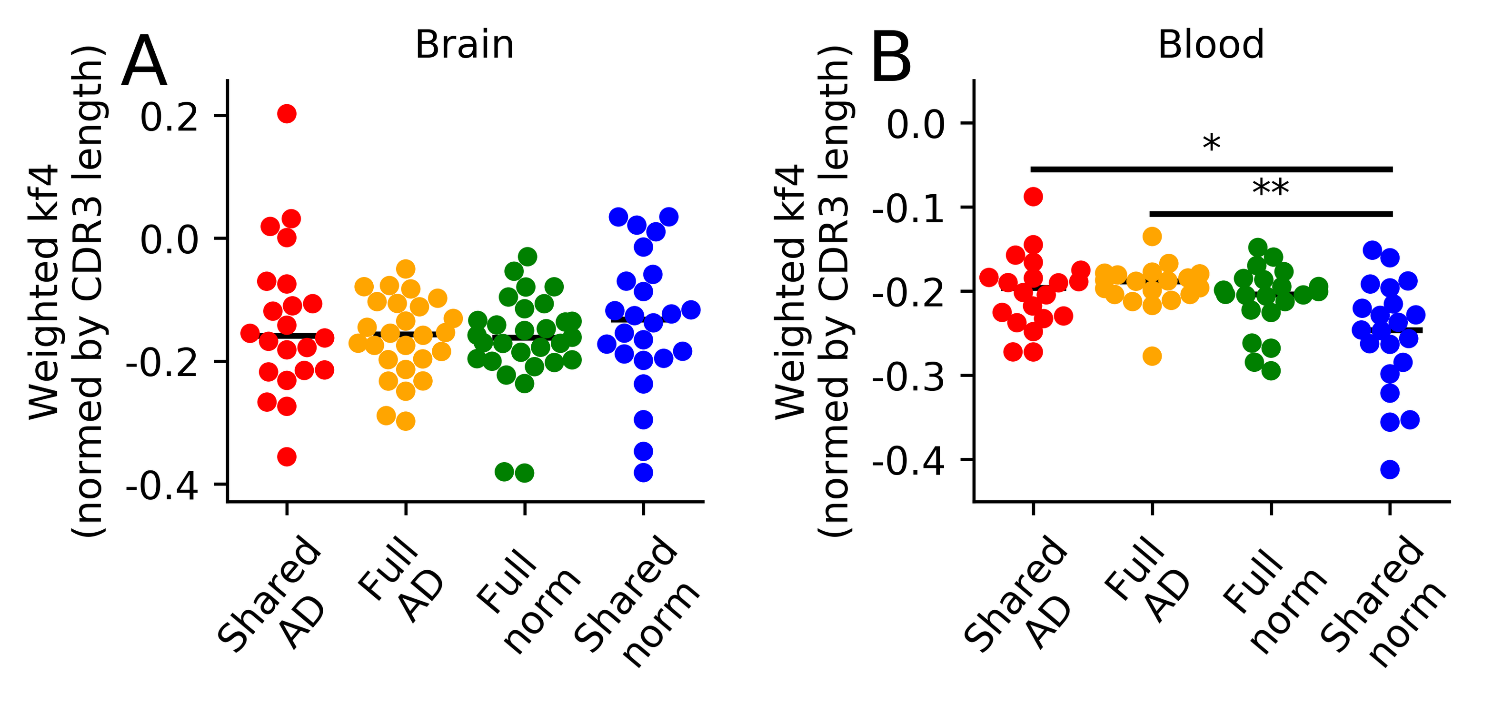


Fig. S6. **Hydrophobicity-related KF4 index analysis of shared and full repertoires.** Comparative analysis of the weighted KF4 index normed by CDR3 lengths between shared AD repertoires (clones found in at least two patients, but not in healthy individuals), full AD repertoires, full norm repertoires, and shared norm repertoires for brain (A) and PB (B) samples. **P* < 0.05, ***P* < 0.01.

# Supplementary Tables

**Supplementary Table 1. Samples**. List of blood and brain tissue samples used to prepare libraries for deep sequencing of the TRG repertoire.

**Supplementary Table 2. V segments.** Comparative analysis of V-segments frequencies in TRG repertoires from brain and PB.

**Supplementary Table 3. Clonotypes.** AD-associated clonotypes observed in brain and blood tissues.

# Supplementary Data

# Supplementary Data Sheet 2. TRG clonosets. Preprocessed CDR3 TRG repertoires.
